# The High-Risk Profile of Selective Growth Restriction in Monochorionic Twin Pregnancies

**DOI:** 10.3390/medicina59040648

**Published:** 2023-03-24

**Authors:** Zoltan Kozinszky, Andrea Surányi

**Affiliations:** 1 Department of Obstetrics and Gynaecology, Danderyds Hospital, 182 88 Stockholm, Sweden; 2 Department of Obstetrics and Gynaecology, Albert Szent-Györgyi Medical School, University of Szeged, 6725 Szeged, Hungary

**Keywords:** monochorionic twin pregnancies

## Abstract

The present review aims to provide a critical appraisal of the sonographic diagnosis and follow-up and to evaluate the optimal clinical management of monochorionic twin pregnancies where one of the twins is complicated by selective fetal growth restriction (sFGR). The classification is based on the umbilical artery (UA) diastolic flow reflecting the outcome. If the sFGR twin has positive diastolic flow (Type I) then the prognosis is good, and it does not require close surveillance. Biweekly or weekly sonographic and Doppler surveillance and fetal monitoring are recommended strategies to detect unpredictable complications in type II and type III forms, which are defined by persistently absent/reverse end-diastolic flow (AREDF) or cyclically intermittent absent/reverse end-diastolic flow (iAREDF) in the umbilical waveforms, respectively. The latest forms are associated with an increased risk of unexpected fetal demise of the smaller twin and 10–20% risk of neurological injury in the larger twin in addition to the overall risk of prematurity. The clinical course can be affected by elective fetal therapy (‘dichorinization’ of the placenta with laser or selective fetal reduction) or elective delivery in the presence of severe fetal deterioration. The prediction of the clinical outcome in complicated cases of type II and III sFGR cases remains elusive. Novel routines in fetal and placental scans in order to predict neurological impairments and unexpected fetal death to optimize the delivery time-point are needed.

## 1. Introduction

Approximately 20% of twin pregnancies are monochorionic (MC), constituting 1 out of 250 pregnancies, and 70–75% of them are monozygotic twins [1,2,3]. Almost all MC twins are monozygotic, which means that both fetuses share a common genotype, genetic growth potential and maternal physiology with their genetically identical counterparts. Furthermore, only a negligible number (~5%) of MC twins are monoamniotic (MA), which represents a reported incidence of 8 out of 100,000 pregnancies comprising 1% of all twin pregnancies [2,4]. MC twins arise due to either an early embryonic cleavage (between day 3 and 7) of a single fertilized egg (diamniotic twins) (DA) [5] or a late cleavage between day 8 and 12 following fertilization of MA twins [6]. About 1 in 5 twin pregnancies are monochorionic diamniotic [1]. Assisted reproductive technology increases the incidence of MC twins due to the fact that in vitro fertilization techniques enhance monozygotic splitting. Based on speculation, assisted hatching and embryo manipulation techniques have a degree of influence on the timing of embryonic splitting twins [6,7]. In addition, iatrogenic MCMA twins may originate from MCDA twins as a result of accidental rupture of the intertwin membranes following invasive prenatal procedures [8].

Generally, twinning gestation poses a higher risk of perinatal morbidity (iatrogenic preterm delivery) and mortality as compared to that of singletons [9,10,11,12] Among twins, chorionicity primarily determines the outcome and more importantly causes MC twins to have perinatal mortality that is nearly triple that of dichorionic (DC) pairs [13]. Additionally, MC twinning is correlated with increased fetal loss, intrauterine demise, and perinatal morbidity and mortality [14]. Congenital anomalies of one of MCDA twins are twice as common as in singleton pregnancies. This is presumably because of the transfusion imbalances or the teratogenic effect of early cleavage. However, aneuploidies are less frequent mainly due to early demise, and the chromosomal anomalies affect both twins [1]. The excess morbidity is predominantly associated with unequally shared placental territories and the almost universal presence of vascular interfetal anastomoses between the twins [15]. In addition, MA twin pregnancy is associated with significantly increased complication, and antenatal and perinatal mortality rates when compared to DC or monochorionic diamniotic (MCDA) pregnancy in general [16,17,18,19].

This review intends to provide a comprehensive critical description of the clinical management and diagnostic follow-up with sonography concerning the complications of MC twins with a focus on sFGR.

### 1.1. Complication Profile of Monochorionic Twins

Overall, one out of three MC twin pregnancies will develop specific complications by way of congenital defects and growth restriction based on placental insufficiency of one or both twins and nearly always present interfetal placental vascular connections encompassing twin-to-twin transfusion syndrome (TTTS), isolated discordant growth, twin anemia–polycythemia sequence (TAPS), twin reversed arterial perfusion (TRAP), or intrauterine demise [19,20,21]. The differential diagnosis of the complications of MC twins is still challenging. This is also on account of the frequent overlap of clinical signs between transfusion syndromes and growth restriction [21].

### 1.2. Selective Fetal Growth Restriction

Selective fetal growth restriction (sFGR) is a condition that occurs in twin pregnancies when one of the fetuses is supplied with insufficient nutrient and oxygen content through the placenta to grow at a normal rate [22,23]. It is diagnosed when the estimated fetal weight (EFW) on a sonographic scan of the growth-restricted twin falls below the 10th percentile, while the other twin is appropriate for gestational age. It occurs in 12–15% of all MC twin pregnancies.

The prenatal diagnosis of FGR is sometimes problematic and it is not possible to find all the cases. Plenty of EFW formulas are available, and some of them are more accurate in normal ranges; however, in the diagnosis of FGR the Hadlock B formula had the best correlation [24]. On the other hand, it is established that all routinely used EFW formulae would overestimate the fetal weight under the normal range; therefore, mild FGR is usually undiagnosed before birth. Specific growth charts are needed in MC twins, which are different from singleton pregnancies [25,26]. It is a prerequisite a significantly high intertwin weight discordance that may exceed 20–25 percent, and it is calculated as the difference between the EFW of the larger twin and the smaller twin divided by the EFW of the larger twin [1,21]. This is of clinical importance when both twins are growth-restricted or twins are normally but discordantly developed and are not classified in this group. Despite the expectations, sFGR can be seen more often in MCDA pregnancies than in MCMA pregnancies, which can be explained by the frequent close cord insertions besides the milder extent of unequal placental sharing and the almost ubiquitous presence of large interfetal arterio-arterial (AA) anastomoses [1,2,17,27,28,29].

The early forms appear in the midtrimester period (below 20 weeks of gestation) and affect 10–12% of MC twins [30], whereas late sFGR is found in 5–6% of MC twins [21,31]. Although the distinct subforms have different pathomechanisms, a varying disproportionate division of the placenta between the MC twins is a common feature. In addition to discordant placental territories, partial functional inactivation of the placenta can contribute to the pathomechanism [31,32,33].

### 1.3. Histopathological Aspects of the Monochorionic Placenta

The pathological feature of the early and more serious form is the variation of the size, types, and number of anastomoses and the distance of the cord insertions, and, hence, the intensity of the placental intertwin blood transfusion varies extensively, presenting a wide range of unique clinical manifestations [21,34]. The weight discordance correlates positively with the disproportionality of placental territory; however, as gestation advances, the corresponding rate alters [15,31,33,35]. Extremely asymmetric distribution of placental territories is often associated with very eccentric or velamentous cord insertion [36], although it is unclear whether velamentous insertion is a mere consequence of the asymmetric displacement of the vascular equator or whether it has any implications in the pathophysiology of growth restriction [34]. However, the incidence of velamentous cord insertion in MA placentas (4%) is lower than that in DA pregnancies (12–20%) [37].

The interfetal vascular anastomoses are randomly distributed between the MC twins, influencing the imbalanced placental blood flow between the fetuses and affecting the clinical course. The main feature of MC twins is the existence of placental anastomoses, which can be AA, veno-venous (VV), or arterio-venous (AV). Vascular placental connections result in bidirectional fetofetal transfusion, a sort of third circulatory system between two fetuses. AV anastomoses have unidirectional flow (from recipient to donor) and are vascular AV connections where a placental cotyledon is perfused by an artery from one fetus and drained to the other one. Blood transport through the intertwin AA and VV anastomoses in either direction, usually opposite to the AV anastomoses (from donor to recipient), may compensate for the imbalances in blood volume or pressure between the twins. AA anastomoses connect the two cords in the placenta, directing from one or two UAs. These types of anastomoses are usually thick, with a diameter of ≥2 mm that allows a larger amount of blood interchange. The smaller twin receives oxygenated blood from its co-twin via placental anastomoses to partially compensate for the placental insufficiency [15,31,33,35]. Furthermore, a larger placental share discordance results in a massive transfusion towards the smaller twin, and, hence, the interfetal anastomotic area, net AV transfusion, and the diameter of AA anastomoses correlate with the placental discordance and a milder clinical outcome. The growth-restricted fetus is supplied with well-oxygenated blood from the ’placenta reservoir’ as protection from acute hemodynamic imbalances. However, this ’rescue transfusion’ does not occur constantly, and may temporarily decrease the blood supply to the brain in the larger twin, leading to severe neurological sequelae [15,31,33,35]. The high incidence of double fetal death in MA pregnancies is possibly related to the superficial connecting vessels (AA or VV anastomoses) in the placenta, which enhances rapid blood transfusion, leading to hypovolemia in the surviving twin [27].

The worse clinical evolution is characterized by large placental discordance with very few intertwin anastomoses [34]. Aside from inadequate placental sharing and vascular anastomotic pattern, ’placental crowding’ (inadequate adhesion site of the placenta and/or insufficient placental volume) might contribute to the relative placental insufficiency of the small twin.

### 1.4. Classification and Management Options

The classification of the subtypes is based on the end-diastolic flow (EDF) in the UA of the growth-restricted fetus that corresponds well to three clinical expressions [20,21]. These Doppler patterns can be observed from the early stage during pregnancy, usually before the 20th week of gestation, remaining unchanged until delivery [38,39]. UA Doppler alterations are secondary to placental insufficiency [40] and are also with distinct intertwin vascular connections [41].

The most frequent subform is type I sFGR fetuses, with an incidence of 29–63.5% [42,43] which are distinguished by constantly positive diastolic UA flow. Type I is characterized by a relatively favorable outcome due to the lack or diminished number of thin AA anastomoses, similar to that in uncomplicated MC twins [44]. The lower degree of intertwin discordance is due to the reasonable compensation of the disproportionately shared placenta function by an appropriate number of large AV anastomotic pattern allowing bidirectional fetal flow interchange. The ratio between the degree of fetal and placental discordance is 1 in uncomplicated MC pregnancies, whereas the corresponding ratio is smaller than expected in type I sFGR pregnancies. Since the grade of growth restriction is low and stable throughout pregnancy, the risk of fetal death of the affected fetus and the parenchymal brain damage of the unaffected twin is reduced (2–4% and 0–4.3%, respectively) [33,35,41]. The course of growth restriction usually does not progress and is not linked to umbilical flow changes. The gestation can be prolonged up to >34 weeks as growth restriction does not progress. Conservative management and regular biweekly or weekly sonographic and Doppler examination follow-up could be a reasonable means of ruling out progression to type II [20,21,30,34] (Table 1).

For the most severe variant (type II) (incidence: 22.4–36.5% of the sFGR) [42,43], a persistently absent/reverse end-diastolic umbilical flow (AREDF) can be registered, and the deterioration of the flow can be utilized in the prevention of the intrauterine demise of the compromised fetus which may occur at high risk. However, the risk of periventricular leukomalacia in the healthy fetus is low, and the gestation might continue only until week 29 as a mean value due to the high risk of in utero death. A severe discrepancy in the intertwin placental territory occurs because of the lack of a vessel net or the limited small interfetal AA anastomoses, ensuring only brief compensation. Although the placental angioarchitecture is similar to that in the uncomplicated MC twins, the fetal weight/placental weight discordance ratio is remarkably low, and the intertwin blood shift attenuates the severity of the placental insufficiency. Placental insufficiency cannot be fully compensated by intertwin blood exchange. Expectant management might lead to the demise of one-third of the sFGR fetuses and even one-fifth of the larger twins, while 15% of the small twins have severe brain damage [45]. Ductus venosus (DV) PI reduced or reverse a-flow in ductus venosus (DV) is identified as a significant predictor of survival [30,42,43]. Besides the DV PI, severe fetal deterioration identified by pathological waveforms in the umbilical vein or reduced biophysical profile might be avoided by elective delivery before 30 weeks. A weekly follow-up is considered if the venous umbilical flow is normal, but more frequent monitoring is indicated when DV PI is elevated (>2 standard deviation (SD)). There is little evidence supporting use of cardiotography (CTG) in cases of prematurity [20,21,30,34]. Fetoscopic laser coagulation might be advocated to protect the larger twin from the death of its co-twin in case of severe sFGR at early gestation, with signs suggesting fetal deterioration. It carries higher obstetric risks and is associated with a high risk of the demise of the FGR fetus in any case due to the surgery interrupting the protective anastomoses. However, the coagulation of the vascular equator is more difficult because there is no polyhydramnios in the amniotic sac of the donor, and it is more challenging to perform than in TTTS [46]. On the other hand, cord occlusion of the deteriorated fetus is a more advantageous optional treatment with expected survival rates for the normal twin ranging from 90–95% [42,47] (Table 1).

The type III subform (48% of all sFGR cases) [20,42] is accompanied by an intermittently absent/reverse EDF in the UA (iAREDF), showing an alternation of phases of positive with phases or AREDF, normally in a cyclical pattern. The oscillatory changes in systolic velocity can be observed in either UA or only one artery, which can change along the umbilical cord due to the presence of the Hyrtl anastomosis. The characteristic feature of this Doppler pattern arises from the transmitted systolic waveforms from the larger into the smaller twin’s cord due to at least one large AA anastomosis. AA anastomoses involve only one UA [30,34] (Table 1).

The apparently benign clinical evolution provides a low risk of hypoxic damage to the FGR fetus and facilitates the gestation until 32 weeks, or in milder form to 34 weeks. The fetal deterioration is unpredictable, with scanning of the DV flow, umbilical vein Doppler, or biophysical profile changes, and elective delivery around 32 weeks is recommended with weekly follow-up if venous Doppler is normal [20,21,34,44]. However, the reported unpredictable fetal loss is approximately 10–15% among the growth-restricted fetuses, while 10–15% of the type III cases are complicated by brain damage of the normal fetuses. To a large extent, unequally shared placental functional allocation (up to 10:1) is compensated by relatively large AA anastomotic vessels improving the survival of the sFGR fetuses and prolonging the pregnancy. AA anastomoses behave as functional AV anastomoses associated with a short distance between placental cord insertion sites, and the fetal weight/placental weight ratio is the lowest in this group (<0.5 on average). The placental territory discordance may be >10 (extremely unevenly divided placenta) and the smaller twin almost entirely depends on the intertwin blood exchange with the larger twin [37]. The AA anastomoses are detrimental due to the risk of lethal acute fetofetal hemorrhagic accidents [20,21], but bidirectional flow of AA anastomoses is reflected by the collision of two opposite systolic waveforms. If the placental cord insertions sites are at shorter distance and the AA anastomoses are large, then more pronounced reversed diastolic waveforms can be traced in the FGR twin. However, the compensating effect of the large AA anastomoses protects against the hypoxic deterioration of the small fetus. The large twin has a hypertrophic cardiomyopathy-like heart with a reactively increased pumping function [48]. These cases are associated with a significantly higher risk of unexpected fetal death of the FGR fetus and white matter injury of the normal-growth twin or concomitant death of the larger twin due to acute interfetal transfusion episodes of large amounts of blood, particularly when AA anastomoses are present [49]. In addition, sFGR may be associated with neurological impairment of the normal-growth twin even if both fetuses are born alive [30]. Endoscopic placental laser therapy is a feasible option in severe forms of type III sFGR cases in previable gestation; however, it is technically more challenging than that in type II due to larger anastomoses [50]. Cord occlusion for the fetus presenting with extreme forms of FGR and early DV flow alterations and/or even laser coagulation of the intertwin anastomoses can be offered to protect the surviving twin from demise or neurological damage [42,50] (Table 1).

Generally, the most severe complications are attributed to the type II forms, but type III sFGR fetuses are also at a high risk of very preterm delivery, intrauterine fetal death of the smaller twin, and neurological sequelae of the twin with normal growth. The severity of the complications is highly related to the grade of growth restriction and its progress, and the gestational age at diagnosis. UA Doppler examination cannot be used as a prognosticator of imminent fetal death, but the manifestation of absent or reverse atrial flow in the DV or abnormal venous Doppler can be used for appropriate timing of delivery. Fetal therapy (laser therapy or selective fetocid) has been proposed for use only in severe cases diagnosed at the <24–26th weeks of gestation or at signs of imminent fetal demise before viability. Type II and III sFGR pregnancies treated with laser therapy or cord occlusion have a higher rate of perinatal mortality and premature birth but a lower rate of morbidity compared with those managed expectantly [1,22]. However, laser therapy has been reported to be associated with substantial mortality rate in the small twin (60.5% and 60–80% in type II and III, respectively [12,34,51,52]. Fetal demise in type III after laser therapy can occur in 32.9–41.0% of FGR fetuses, but the survivors were free from major neurological complications [22,34]. Cord occlusion is more beneficial in terms of mortality of the small twin, and almost all larger twins survive in early and severe type III cases [22].

The clinical pattern of the growth-restricted twin differs prominently from that of singleton or in DC pregnancies where there are two entirely separated chorionic ’units’. sFGR fetuses in MC pregnancies show a remarkably longer latency between the onset of the alteration of the umbilical flow and delivery (10–14 weeks) compared with the 3–6 weeks reported in singletons with FGR with deteriorated umbilical blood flow [38,39]. The relatively high risk of neurological injury of the larger twin in type II and type III is suspected due to non-lethal acute blood exchange episodes via transplacental anastomoses, which are reflected by the temporary intermittent umbilical flow [44,49,50,53].

The umbilical Doppler ultrasound patterns of the smaller (recipient) twin are not always stable, and a considerable proportion of the sFGR changes its classification. The highest risk of fetal death is encountered in pregnancies with type I sFGR that evolves type II or III or type II subforms pass into type III subform [43,54]. Nonetheless, the absent EDF in the umbilical flow at 16–18 weeks is a physiological and not a pathological sign [22].

A slowly progressive intertwin discordance may contribute to the late sFGR of the compromised twin, while normal growth can be described for the healthy fetus. Late sFGR has always normal EDF and can be demonstrated solely in the third trimester (>28 weeks of gestation) and is not observed in the second trimester. The rate of late onset sFGR was reported to be 6.3% among MC pregnancies. A remarkably benign clinical outcome with a low risk of intrauterine fetal death (8%) is associated with this group. An underlying placental insufficiency results in a progressively developing weight discrepancy reaching a mean of 30%. The reported frequency of TAPS was 38%, which suggests the significance of the monitoring of peak systolic velocity (PSV) of the middle cerebral artery (MCA) and pulsatility index (PI) in DV for close timing of pregnancy in these cases [12]. Long-term sequelae in the late onset sFGR are yet to be determined [31,34,55].

sFGR occurs in 10–15 percent of DC twins, but unlike MC pregnancies, DC twins have entirely separate fetal-placenta blood circulations, and thus the larger twin is unaffected [56,57].

## 2. Other Complications in Monochorionic Twinning: Twin-to-Twin Transfusion Syndrome (TTTS), Twin Anemia Polycythemia Sequence (TAPS), Twin Reversed Arterial Perfusion (TRAP), and Non-Specific Amniotic Fluid Discordance (AFD)

A detailed description of other complications than sFGR in MC twins is beyond the scope of this review; however, a short interpretation of the MC-related unique complications is provided. The sFGR implicates separate pathomechanisms from TTTS [58]), TAPS [59,60], TRAP [42,61,62], and isolated amniotic fluid discordance (AFD) [21,63] leading to different sonographic follow-up and therapy/management requirements. The placenta has different types of anastomoses and angioarchitecture and is divided differently between the twins in these pathological conditions [28].

TTTS is a serious condition that occurs in about 10–15% of monochorionic twin pregnancies usually before 26 weeks [64,65]. In most cases, the blood flow is unevenly distributed throughout the unidirectional AV/VA anastomoses in the placenta. The donor transfers blood to the recipient, resulting in hypovolemia in the donor and hypervolemia in the recipient. Excess blood flow from the donor to the recipient depends on the number/size of anastomoses, finally causing the death of both fetuses if no surgical procedure takes place [21,66]. TTTS is presumably related to the lack of circulatory balancing AA anastomoses [27].

Fetoscopic laser coagulation is the optimal therapy to considerably improve the co-twin prognosis by clogging abnormal anastomoses [67]. Coagulating the placental vascular equator after selective coagulation of the anastomoses before 26 weeks increases the perinatal outcome. The imbalance in AV anastomoses is often combined with other factors such as fetal weight discordance/restriction, relatively slow placental growth, cord insertion deficits, or other fetal malfunctions (e.g., cardiac defects), which may trigger the TTTS [68]. Approximately 60% of the TTTS cases are combined with sFGR, which is significantly more common among the donor twins, increasing its perinatal risk [21,42,43,69]. TTTS can develop prior to or after an initial diagnosis of sFGR. Coexisting sFGR prior to fetoscopic laser surgery might lead to a decreased donor perinatal survival compared to that in the TTTS-only group; however, the long-term outcome is not different [42,43]. TTTS manifestations rarely occur in MCMA pregnancies compared to MCDA twins due to compensating AA anastomoses [35,70,71].

TAPS appears in 3–5% of MC twins, usually in the third trimester (mainly after 26 weeks) [72]. Postnatal diagnosis of TAPS requires a difference of ≥8 g/dL in hemoglobin [1]. Unbalanced transfusion arises through a small and superficial interfetal AV anastomosis net with significantly less intensity in TAPS than in TTTS [73] ), and the chronic subtle transfusion results in anemia–polycythemia sequence with good prognosis. Incomplete laser coagulation of the chorionic plate anastomoses in TTTS cases might lead to a rapidly progressing (within 1–4 weeks) TAPS in around 2–6% of cases, requiring therapy after determining the pathological value at Doppler measurement/prehydropic case [74].

sFGR and TAPS can be individually observed or can co-occur in MC twin pregnancy. The placental anastomoses of sIUGR with TAPS are typically small. sIUGR with TAPS had smaller differences in placental share and larger distances between umbilical cord insertions. Late sFGR, when the growth rate reduces gradually, could be accompanied by abnormal blood flow in the umbilical cord artery during the diastole. This allows the donor fetus to transfuse blood chronically to the recipient fetus through these anastomoses, leading to anemia in the donor and hypervolemia in the recipient fetus sIUGR with TAPS had smaller differences in placental share and larger distances between umbilical cord insertions [75].

TAPS must be regularly screened by means of routine middle cerebral artery (MCA) Doppler ultrasound examination (MCA Vmax >1.5/<1 MoMs in the twins) since it has no other manifestations besides the DV PI measurement [76]. If untreated, severe anemia of the donor fetus might lead to cardiac decompensation and hydrops. TAPS might complicate late onset sFGR and expectant management with close surveillance of the peak systolic velocity of the MCA or intrauterine transfusion are also acceptable clinical management modality [72].

In TRAP, a parabiotic twin has an absent or non-functioning cardiac system and receives blood from the normally developing twin, often referred to as the ‘pump twin’. Because one heart is pumping blood for both twins, the condition places an enormous demand on the heart, putting the pump twin at risk for cardiac failure. TRAP can develop as a complication of TTTS laser treatment of TTTS. In combined cases, the formation of sFGR is independent of TRAP development [77].

AFD is usually defined as a difference in amniotic fluid volumes in a twin pregnancy. An AFD ≥ 4 cm cutoff is associated with a significantly increased risk of the development of TTTS (70%) [68,78]. 

## 3. Sonographic Concerns

### 3.1. Screening during the First and Second Trimester

Accurate diagnosis/identification of chorionicity and amnionicity is of paramount importance [17,21,79]. The identification of chorionicity can even be performed prior to the 10th week of gestation [80]; however, late detection of chorionicity can occur at the 11 to 14 weeks, demonstrating the presence of the T-sign (direct binding of the two thin amniotic membranes) as an ultrasound sign of monochorionicity. The sensitivity and specificity of sonographic diagnosis of monochorionicity were 81.1% and 96.0% based on T-sign [81,82,83]. Structural anomalies are reported at rates up to 25% in MCMA twins [79], and weight discordance is often concomitant since commonly only one fetus is compromised [17,79]. Fewer MCMA twins with congenital anomalies are linked to underlying chromosomal abnormalities (4%) than their DC or MCDA counterparts [84]. The high incidence of anomalies is considered to be due to the delayed embryonic splitting and the placenta-derived transfusion imbalance [17,27,57]. Severe sFGR with flow anomalies in early gestation, irrespective of major abnormality, is commonly treated by fetoscopic umbilical cord transection and administering selective feticide to avoid further cord accident/entanglement and to increase the survival chance of the healthy twin [18,85]. This clinical practice is evident even in the case of the demise of the severely restricted fetus, both in MCMA and MCDA settings [18,86].

### 3.2. Follow-Up of the Fetal Growth in the Second and Third Trimester

The lowest birth weight and gestation at birth can be reported in MC twins compared to singleton or DC pregnancies. Growth-restricted fetuses (birth weights below the 5th percentile) are more often observed in MCDA pregnancies (37.8%) than in MCMA pregnancies (33.3%) where at least one fetus is born alive, and only the former rate is significantly higher compared to DCDA twins (31.2%). However, both fetuses were discovered to be growth-restricted in most of the pregnancies in these cases [2]. Furthermore, monoamniocity carries a higher risk of cord entanglement, and the two umbilical cord insertions are most commonly close together [17]. Twin fetuses’ growth is usually delayed during the third trimester (>30–32 weeks of gestation) compared to singletons, which has been attributed to ‘placental crowding’ and the more frequent anomalous abnormal umbilical cord insertion [87]. It is recommended that a distinguished twin growth curve pattern should be applied in the third trimester, which differs from the singleton percentiles because fetal growth starts to decelerate from 32 weeks of gestation [26,88]. 

### 3.3. Secondary Sonographic Features

The perinatal mortality of MCMA twins is between 30 and 40% [2]. The overwhelming majority of fetal death occurs in the first or second trimesters due to congenital anomalies or termination of pregnancies based on congenital anomaly, and the risk of loss is nine times higher than that among DC twins [17,89]. In non-anomalous MCMA pregnancies, cord-related accidents/entanglements and/or acute loss of fetal hemodynamic equilibrium due to large placental anastomoses are often associated with fetal death [17,79]. The risk of the demise of both fetuses, following single fetal death in MC pregnancies, is high. The surviving fetus may lose a part of its circulation through the large AA anastomoses, which may lead to ongoing hypotension, multiorgan hypoperfusion, and consequent ischemic brain damage/loss of a larger twin which can be prevented by fetoscopic cord transection or laser ablation of the anastomoses [1,17]. A fetal brain MRI at least 3–4 weeks following the co-twin death is advised to detect brain injury and to improve postnatal neurodevelopment [90].

The lowest birthweight and gestation at birth can be observed in MC twins compared to DC twins [2]. Determination of the number of yolk sacs can also be considered as an adjunct method in the identification of MA status [91,92]; however, one-third of MCMA pregnancies have double yolk sacs [91]. Although the incidence of TTTS is reported to be lower, cord entanglement or knotting of the umbilical cords and a consequent fluctuant fetal position are frequently present in twins with a single amniotic cavity, which makes the labeling of the twins difficult, particularly if growth discordance is not present. The cord insertion may be helpful in the labeling process, but cord entanglement makes the identification process difficult [17]. Placental three-dimensional power Doppler vascularization indices seem to be appropriate for predicting birth weight in MCDA and DCDA normal twin pregnancies [32]. Diminished vascularization indices are characteristic of placentas in MC twins complicated by all subforms of sFGR, while placental perfusion was significantly impaired only in type II sFGR fetuses [93]. No study dedicated to placental blood perfusion in MCMA twins has been conducted so far.

### 3.4. Comparison of the Surveillance and Management Guidelines

Foremost, first and early second trimester sonography should be applied in aiding the determination of amnionicity/chorionicity [80,82], major congenital anomalies in MC pregnancies [94], and conjoined twins in the MA setting. Furthermore, screening during weeks 13–28 is advocated to detect further anomalies and treat serious complications amenable to fetal therapy (TTTS, early sFGR type II or III) and in weeks 29–37 for early detection of complications in the third trimester (late sFGR, both fetuses with FGR, TAPS, and TRAP) [21]. Late complications are less frequent but usually progress less precipitously.

Complicated cases where sFGR is combined with TTTS, which deteriorates the condition of the donor twin urgently, require immediate laser therapy, so the first step in the diagnostic algorithm is to assess TTTS. When routine sonography is performed, TTTS and sFGR should be assessed first, whereas TAPS and particularly AFD are arbitrary phenomena [21]. Primarily, the diagnosis of the TTTS is based on the marked discordance in AF volume, which means a combined presence of the deepest vertical pocket of ≥8 cm in one sac and ≤2 cm in the other, regardless of the gestational age at detection. Secondly, a serial ultrasound assessment of the fetal biometric parameters every second week permits the detection of sFGR starting at 16 weeks of gestation through measuring the discordance. Thirdly, MCA PSV measurement in the fetuses may be used to evaluate the TAPS sequence [88,95,96]. sFGR can progress into TTTS and TAPS and type I may progress to type II or III that may decrease the survival. Type II without signs of imminent death or type III can be managed expectantly and laser therapy would deteriorate the anastomoses that alleviate the impact of sFGR [1].

Generally, there is no evidence of optimal monitoring of MC pregnancies, but most studies recommend a sonographic follow-up at least biweekly/weekly, since TTTS can appear even days after an examination with conclusion of normal values [17,21,97]. The commencement of intensive surveillance decreases the potential risk of fetal death [97,98]. To detect early MC-related complications, sonographic follow-up measuring fetal biometry, umbilical and middle cerebral artery, and DV by Doppler examination and assessment of amniotic fluid volume and fetal urinary bladder can be applied from the 16th week of gestation every second week up to the delivery in MCDA pregnancies [21,31]. Similarly, a complete scan with estimation of the fetal weight can be introduced biweekly from the detection, and a weekly Doppler flowmetry might be recommended after the 16th week of gestation in MCMA pregnancies. A standard complete ultrasound evaluation including additional scan modalities (including DV flow investigation by Doppler ultrasound examination) is recommended when a complication is detected, and repeat exams are recommended according to the extent and type of complication [21,31].

Accordingly, a simultaneous follow-up of the fetal heart monitoring (computer-task CTG) from viability (from 24th weeks of gestation) or later (from 28th weeks) and onwards is often integrated into the twin monitoring system [1,97,99]. However, fetal monitoring by computerized or conventional cardiotocographic evaluation does not indicate the fetal deterioration in sFGR pregnancies [100]. Although weight discrepancy is often related to adverse perinatal outcomes, its prognosticating effect on acute fetal events is poor [98] and the extent of the growth restriction usually does not determine the optimum timing of the delivery. The appearance of the diastolic notch in the uterine artery [101], or absent/reversed EDF [8,48] in the umbilical artery waveform due to entanglement occurring transiently or constantly, also has only limited value in the decision process of delivery. Fetal heart rate tracing is usually just an additional modality, but persistent prolonged decelerations or persistent tachycardia will frequently be considered as a prompt delivery criterion [8]. 

The surveillance intensity and approach vary in the studies with no obvious consensus [17,21,99,102,103] in MCMA pregnancies. Controversial results revealed no advantage of either of the surveillance methods deriving from elective inpatient or outpatient care ranging from continuous and several times daily fetal monitoring to lacking alternate daily surveillance. An increased risk of intrauterine fetal demise can be observed in outpatient care compared to inpatient care (7% vs. 3%) according to a systematic review [99], though acute ischemic events cannot be predicted even with intensive surveillance [17,79,99].

## 4. Timing of Pregnancy

It is of paramount importance that timely delivery is mainly guided by avoiding fetal loss in MC pregnancies [103]. Elective preterm delivery is usually performed when the risk of fetal loss upon continuing the pregnancy outweighs the risk of prematurity [12,17,70,104]. Generally, the prospective risk of fetal death after the 32nd week of gestation (4%) outweighs the risk of neonatal death (1%) and non-respiratory prematurity complication at delivery at 32 weeks in MCMA twins [70,99,103]. Cesarean delivery at 32–33 weeks of gestation carried out by experts due to cord entanglement during labor is suggested by the practical guidelines in MCMA pregnancies [17,70,88,95,96,105,106]. However, in a recent small retrospective study by Chitrit et al. [107], vaginal deliveries were observed as a safe delivery mode at 33 weeks of gestation for MCMA twins.

However, the timing process of delivery carries uncertainty and depends on many factors: flow parameters (UA Doppler flowmetry, DV PI or atrial flow pattern, umbilical vein flow, or PSV of the MCA), the extent of the weight discordance and the growth restriction, the gestation at diagnosis, the available technical factors, and finally the parents’ preferences, keeping in mind that a substantial proportion of sFGR twins, particularly in type II or type III, have very low birthweight due to prematurity [12]. 

## 5. Pregnancy and Neonatal Outcome

MC twinning poses a greater perinatal risk compared to DC gestations [22,108,109,110]; however, weight discordance belongs to the major determinants of perinatal outcome in twin pregnancy [9,10,11,12,17]. Moreover, MC twins had an inherent risk of prematurity, and gestational age is the main determinant of perinatal outcome. Monochorionicity accounts for 2- and 4-times higher rates of perinatal mortality than in DC twins and in single pregnancies, respectively [18]. Neurological sequelae are 4–5 times as high as in DC pregnancies and therefore 25–30 times as high as in singletons [108,109].

The presence of one of the growth-restricted twins has a critical influence on the perinatal outcome [9,10,11,12,17]. Furthermore, early onset of the sFGR is raised as a significant concern [31] and coexisting TTTS has no significant impact on the perinatal outcome. Earlier detection of extreme type II and type III cases with higher DV PI is significantly associated with increased risk of poor prognosis. Pregnancies complicated by early and severe type I sFGR pregnancies with large weight discordance are at higher risk of perinatal demise at an earlier gestational age. These pregnancies may benefit from increased surveillance and/or operative treatment [43].

The duration of pregnancy is the longest for sFGR type I cases (between 33.0 and 36.0 weeks of gestation) compared to type II (between 27.6 and 32.4 weeks) and type III cases (between 28.3 and 33.8 weeks). Accordingly, the lowest intrauterine demise (0–4%) [43,111] and cerebral injury (0–2%) [43,45,111] ( are shown among the smaller twins in the type I group than those observed among the affected sFGR twins in type II (up to 40% and 30%, respectively) and type III (up to 23% and 33%, respectively). Neonatal mortality is up to 10% in type I, up to 38% in type II, and up to 17% in type III [43,111]. Moreover, high neonatal mortality of up to 17% was reported among type III growth-restricted twins [43,111]. sFGR type II and type III, which are typically characterized by progressive deterioration, are particularly at risk of cerebral injury, which could be a consequence of the prematurity and the abnormal in utero flow, representing an increased risk of long-term neuro-developmental impairment [112].

## 6. Conclusions

Generally, type I is characterized by good perinatal outcome and is recommended to be managed expectantly. The adverse perinatal outcome in severe type II and III cases raises concerns on the necessity of further prognostic markers. Laser placental coagulation therapy is the suggested treatment for pregnancies complicated by sFGR with coexisting TTTS. However, the elimination of the acute interfetal transfusion imbalances by laser surgery in isolated sFGR to improve the perinatal outcome and to avoid the potential long-term neurological consequences for the cotwin is still to be addressed. The adequate prediction of the risk of transfusion imbalances may assist in better understanding the risk of unpredictable fetal death in sFGR fetuses.

## Figures and Tables

**Table 1:** Characteristics of the subforms of the monochorionic twins with selective fetal growth restriction.

Subform	Ultrasound Characteristic	Placenta Histology	Clinical Course
Type I	Both twins have normal UA flow	Unequally shared placenta. No or small AA anastomoses.	Good prognosis with very low risk of IUFD or neurological damage. Delivery at 33–36 weeks
Type II	The larger twin has normal flow in the UA, whereas the small twin has intermittently absent and reverse EDF in the UA permanently	Severe placental territory imbalance. Small vessel net and exists just a few small AA anastomoses compensate only for short time.	The most severe cases. High risk of deterioration or IUFD of the FGR twin, low risk of neurological sequelae of the larger twin. Delivery at 27–32 weeks usually
Type III	Cyclic absent and reverse EDF in the umbilical artery in the small twin and norm flow in the larger twin	A large discrepancy in the intertwin placental territories. One large AA anastomosis compensates well for the territorial imbalance.	Intermediate prognosis: Low risk of hypoxic ischemia and 10–15% risk of unpredictable IUFD of FGR twin; up to 15% risk of brain injury in normal co-twin. Delivery at 28–34 weeks.

UA: umbilical artery, EDF: end-diastolic flow, AA anastomoses: arterio-arterial anastomoses, IUFD: intrauterine fetal death, FGR: fetal growth restriction.

## Data Availability

Not applicable.

## Abbreviations

AA	arterio-arterial
AFD	amniotic fluid discordance
AGA	appropriate for gestational age
AV	arterio-venous
CTG	cardiotocography
DC	dichorionic
DV	ductus venosus
EFW	estimated fetal weight
FGR	fetal growth restriction
MA	monoamniotic
MC	monochorionic
MCDA	monochorionic diamniotic
MCMA	monochorionic monoamniotic
PI	pulsatility index
PSV	peak systolic velocity
SD	standard deviation
sFRG	selective fetal growth restriction
TAPS	anemia-polycythemia sequence
TRAP	twin reversed arterial perfusion
TTTS	twin-to-twin transfusion syndrome
US	ultrasound
VV	veno-venous
